# Artificial Intelligence-Assisted Transcriptomic Analysis to Advance Cancer Immunotherapy

**DOI:** 10.3390/jcm12041279

**Published:** 2023-02-06

**Authors:** Yu Gui, Xiujing He, Jing Yu, Jing Jing

**Affiliations:** 1 Laboratory of Integrative Medicine, Clinical Research Center for Breast, State Key Laboratory of Biotherapy, West China Hospital, Sichuan University and Collaborative Innovation Center, Chengdu 610041, China; 2 School of Pharmacy, Chengdu University of Traditional Chinese Medicine, Chengdu 611137, China

**Keywords:** artificial intelligence, transcriptomic analysis, immunotherapy, tumor microenvironment, immune-related adverse events, drug resistance, drug target discovery

## Abstract

The emergence of immunotherapy has dramatically changed the cancer treatment paradigm and generated tremendous promise in precision medicine. However, cancer immunotherapy is greatly limited by its low response rates and immune-related adverse events. Transcriptomics technology is a promising tool for deciphering the molecular underpinnings of immunotherapy response and therapeutic toxicity. In particular, applying single-cell RNA-seq (scRNA-seq) has deepened our understanding of tumor heterogeneity and the microenvironment, providing powerful help for developing new immunotherapy strategies. Artificial intelligence (AI) technology in transcriptome analysis meets the need for efficient handling and robust results. Specifically, it further extends the application scope of transcriptomic technologies in cancer research. AI-assisted transcriptomic analysis has performed well in exploring the underlying mechanisms of drug resistance and immunotherapy toxicity and predicting therapeutic response, with profound significance in cancer treatment. In this review, we summarized emerging AI-assisted transcriptomic technologies. We then highlighted new insights into cancer immunotherapy based on AI-assisted transcriptomic analysis, focusing on tumor heterogeneity, the tumor microenvironment, immune-related adverse event pathogenesis, drug resistance, and new target discovery. This review summarizes solid evidence for immunotherapy research, which might help the cancer research community overcome the challenges faced by immunotherapy.

## 1. Introduction

Cancer immunotherapy, especially immune checkpoint blockade, has been one of the most important therapeutic approaches, appearing to have unprecedented clinical efficacy. Immune checkpoint inhibitors (ICIs) show a powerful therapeutic effect in treating patients with cancer by removing the brake on T-cell activation [1]. Many clinical studies have reported on the use of ICIs [2,3,4]. Targeting programmed death-1 (PD-1) pathway is used to treat patients, showing significant improvement in overall survival and progression-free survival [5]. Antibodies blocking cytotoxic T lymphocyte antigen-4 (CTLA-4) are also widely used in the treatment of various cancers. Furthermore, the combination of anti-CTLA-4 and anti-PD-1/PD-L1 ICIs has greater advantages in survival indices than single-ICI immunotherapy [6].

Although great progress has been made in checkpoint immunotherapy, the low response rates and acquired resistance greatly limit the clinical applications of immunotherapy [7]. Moreover, immunotherapy response is often accompanied by therapeutic toxicities, collectively referred to as immune-related adverse events (irAEs), which seriously hinder the sustainability of ICI treatments [8]. Therefore, intense interest exists in better understanding the mechanisms of immunotherapy response and irAE pathogenesis. It is essential to characterize tumor heterogeneity and the tumor microenvironment.

In recent decades, transcriptomic analysis has spread rapidly in cancer research. It has brought remarkable insights in the fields of cancer immunotherapy. The revolution from bulk RNA-seq to scRNA-seq has made transcriptomic analysis more accurate and powerful. Millions of transcriptomic profiles of individual cells have deepened the understanding of cancer heterogeneity and the tumor microenvironment [9]. However, a large amount of transcriptome data also challenged us to believe that conventional methods were unsuitable to comprehensively analyze these data, especially scRNA-seq data. Researchers need more efficient algorithms to handle high-dimensional transcriptomic data. Fortunately, AI adequately meets the growing demand for transcriptomic analysis. AI approaches can improve discovery and decision-making with little human intervention. Applying AI-based frameworks can also help us mine hidden information and obtain more valuable results. In addition, AI can be applied to batch effect removal and cross-platform data integration [10]. The AI-assisted transcriptomic analysis actually accelerates the unveiling of tumor heterogeneity and the microenvironment and improves the prediction of immunotherapy response.

AI generally refers to the combination of big data and algorithms to build models used to solve complex problems and make decisions with little human intervention. It mainly includes machine learning (ML) and deep learning (DL), which have been playing increasingly important roles in mining transcriptome profiles [11]. ML is dedicated to improving the system’s performance by constantly computing. Major popular ML algorithms include random forest (RF), support vector machine (SVM), least absolute shrinkage and selection operator (LASSO) regression, and many other algorithms. DL is one of the most important branches of ML. Commonly used DL algorithms include convolution neural networks (CNNs), autoencoder (AE), generative adversarial networks (GANs), and so on [12]. AI techniques have accelerated the application of precision medicine, especially in the prediction of therapeutic responses and the outcomes of patients. In particular, AI-assisted transcriptomic analysis has provided us with promising solutions to overcome the limitations of cancer immunotherapy, such as irAEs, low response rates, and acquired resistance. For instance, accurate immune signatures and state-of-the-art algorithms have been established for the dissection of tumor heterogeneity and microenvironment as well as the identification of key targets [13,14]. Specifically, AI-based methods or frameworks improved the identification of cell types and status, providing valuable information to accelerate the unveiling of tumor heterogeneity and the tumor microenvironment [15]. They also help us to identify determinators that regulate irAE pathogenesis or drug resistance [16]. Moreover, AI-assisted transcriptomic analysis can accurately capture features or gene signatures, which is instrumental in predicting immunotherapy responses and discovering novel targets [17]. In this review, we summarize new meaningful findings in cancer immunotherapy based on AI-assisted transcriptomic analysis, with an emphasis on tumor heterogeneity, the tumor microenvironment, irAE pathogenesis, drug resistance, and new target discovery.

## 2. Artificial Intelligence Fosters the Application of Transcriptomics in Cancer Research

**Figure 1:**
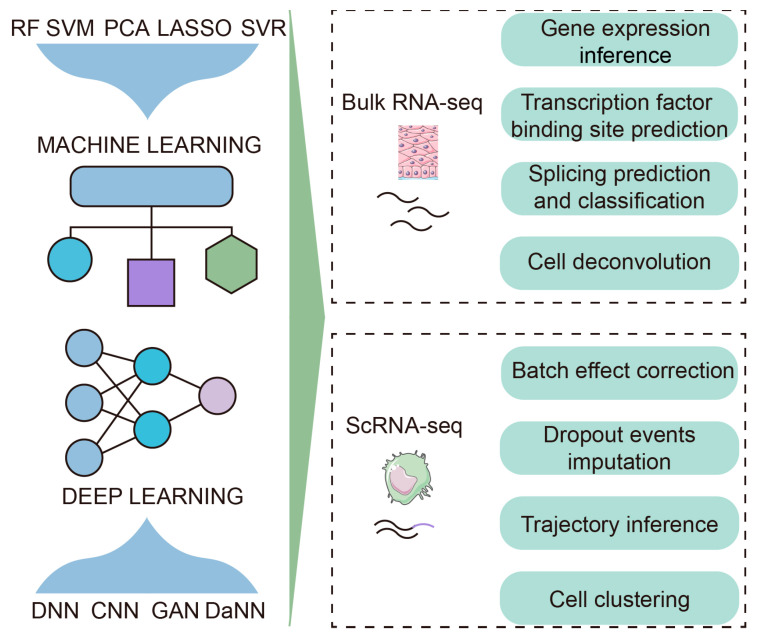
The applications of AI techniques in transcriptomics. AI algorithms have extensively promoted the development of transcriptomics. Numerous cutting-edge bioinformatics frameworks have been established to perform specific tasks, such as gene expression inference, transcription factor binding site prediction, splicing prediction, cell deconvolution, batch effect removal, missing imputation, trajectory inference, clustering, and more. RF, random forest; SVM, support vector machine; PCA, principal component analysis; LASSO, least absolute shrinkage and selection operator; SVR, support vector regression; CNN, convolutional neural network; DNN, deep neural network; GAN, generative adversarial network; DaNN, domain-adversarial training of neural networks.

As an essential part of next-generation sequencing, RNA-seq makes it possible to provide a plentiful supply of transcriptome data of cells or a single cell [18,19]. At the early stage of RNA-seq technology development, most transcriptomic profiles were generated by bulk RNA-seq which provides average gene expression values [20]. Upstream and downstream analyses of these data rely more on arithmetic methods (e.g., differentially expressed gene analysis and gene set enrichment analysis). Currently, many novel and advanced AI-based methods have recently been developed to retrieve more information from bulk RNA-seq data. AI-based computational methods have been widely used in different areas of transcriptomic analysis, such as gene expression, alternative splicing, transcription factor binding, and cell deconvolution [12] (Figure 1). Furthermore, applying AI algorithms in the transcriptomic analysis allows a more accurate identification of the disease state and stage of cancer and accelerates the development of precision medicine.

Tumor tissues are complex ecosystems whose precise characteristics are still masked by bulk RNA-seq despite the advanced algorithms used. Thus, scRNA-seq techniques have emerged as powerful tools to dissect tumor tissues at a high resolution with the advent of precision medicine [21]. This enables researchers to address more complicated biological questions, such as how cells interact with each other in the tumor microenvironment, what happens to cells during cancer progression, and how anticancer agents impact the infiltration of immune cells. At present, scRNA-seq technologies have been widely used in oncology research. Analyzing transcriptome data obtained from scRNA-seq requires AI assistance. Specifically, researchers can hardly solve some problems without AI-based methods, such as high noise levels and missing data [10,12] (Figure 1). In particular, integrating scRNA-seq data from multiple sources may lead to unavoidable batch effects. However, in scRNA-seq experiments, it is difficult to circumvent the batch effect because cells from one condition are typically captured and prepared independently of cells from another condition [22]. To address these limitations, many methods have been launched [23,24,25]. These tools have been successfully applied to denoising, dropout imputation, and batch effect removal. For example, scDetect combines scRNA-seq data and the k top scoring pairs (k-TSP) algorithm to minimize the batch effect and accurately classify single cell types [26]. iMAP based on GANs and deep autoencoders integrates scRNA-seq data from multiple sources to remove batch effects and identify batch-specific cells [27]. Furthermore, AI approaches are also applicable in other unique tasks, such as dimension reduction, clustering, trajectory inference, and more. One of the popular dimension reduction algorithms is t-distributed stochastic neighbor embedding (tSNE), which is frequently used in cluster analysis to better illustrate tumor heterogeneity from scRNA-seq data [28]. Traditional unsupervised learning clustering algorithms can hardly eliminate unwanted technical variations and correct batch effects. The application of AI methods has extensively promoted the development of single-cell omics technology, which in turn inevitably leads to the substantial improvement of data analysis methods. In recent years, scRNA-seq has made remarkable achievements in cancer immunity research, with profound significance in cancer immunotherapy.

To date, high-throughput sequencing technologies have made thousands of transcriptome profiles available. The high heterogeneity and complexity of transcriptomic data is the major challenge that hinders researchers from gaining novel scientific insights [29]. As transcriptome data become larger, the need for AI algorithms is even more evident. AI can separate noise from the true biological signal and provide practical solutions to disentangle the heterogeneity of complex transcriptomes in cancer. Moreover, AI approaches, especially ML, show extreme power in integrating multi-omics data to deliver actionable knowledge [30]. Currently, AI approaches have been pivotal in exploiting the big data generated by omics technologies.

## 3. New Insights into Cancer Immunotherapy Based on AI-Assisted Transcriptomic Analysis

**Figure 2:**
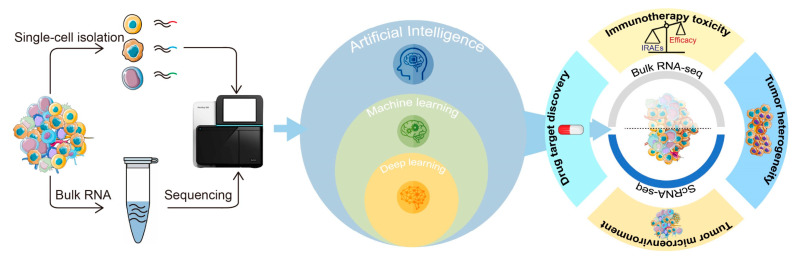
The applications of AI-assisted transcriptomics in cancer research. Transcriptomic technologies have become powerful tools to profile the cancer transcriptome at both bulk and single-cell levels. Currently, AI approaches have been pivotal in exploiting the big data generated by transcriptomic analysis. In particular, the application of machine learning and deep learning in the field of transcriptomic research is rapidly growing. AI-assisted transcriptomics has been frequently utilized to investigate tumor heterogeneity, the tumor microenvironment, novel target discovery, and immunotherapy toxicity.

Although immunotherapy has achieved durable anticancer activity in patients, immunotherapy resistance and toxicity usually occur and curtail its application in clinical practice [31]. Thus, it is critical to explore the determinants driving therapeutic response, drug resistance, and adverse effects. In recent years, AI-assisted transcriptomic analysis has been frequently used to decipher tumor heterogeneity and the tumor microenvironment, determine cellular status, and interrogate the underlying mechanisms of drug resistance and adverse effects (Figure 2).

### 3.1. Tumor Heterogeneity

Intratumor heterogeneity (ITH) is a key factor contributing to patient outcomes and immunotherapy response [32,33]. To determine the ITH level, Sung et al. utilized ML-based approaches to predict ITH using transcriptomics data. They demonstrated that their approach could distinguish tumor samples with high ITH levels from those with low levels and identify transcriptomic markers associated with ITH [34]. To better investigate the heterogeneity of the ICI response, Zeng et al. developed an ML framework based on nonnegative matrix factorization (NMF) to identify factors influencing the ICI response in TCGA samples. They found that ubiquitin E3 ligases may be potential regulators of ICI response [35]. Another group applied the NMF algorithm to transcriptome profiles from ovarian cancer (OV) cell lines. As a result, these cell lines were segregated into five clusters whose histological subtypes and mutational landscape were concordant with clinical cohorts and supported the classification of new model systems of OV subtypes [36].

Bulk RNA-seq data conceal ITH, while scRNA-seq techniques can capture the genetic and molecular characteristics of ITH across diverse stages of tumor development. The analysis of scRNA-seq data highly depends on AI algorithms. The demand for the development of precision medicine requires accelerating the application of scRNA-seq in cancer research. Thus, many cutting-edge bioinformatics frameworks were constructed using ML and DL algorithms to speed up the analysis of scRNA-seq data, such as Seurat [37] and Scanpy [38]. In the case of lung adenocarcinoma (LUAD), scRNA-seq data from 52 freshly resected lung specimens were obtained and Seurat was used to decipher the characterization of epithelial cell lineages in different histologic stages of LUAD [39]. This study uncovered a unique cell population closely resembling alveolar type 2 cells (AT2), termed AT2-like cells, which emerged in the early stages of LUAD and contributed to cellular heterogeneity in LUAD progression. A similar workflow was employed to explore the heterogeneity of epithelial cells in OV. They found that a pivotal malignant epithelial cluster, PEG10^+^ embryonic malignant epithelial (EME) cells, belonging to an intermediate stage of the embryo-to-tumor process, was associated with poor prognosis. Moreover, cell-specific marker genes of PEG10^+^ EME could well discriminate which patients would benefit from immunotherapy [40]. In the case of acute myeloid leukemia (AML) noted for ITH, researchers combined scRNA-seq and genotyping to characterize AML ecosystems and developed RF-based ML classifiers to predict AML cell types and distinguish malignant and normal cells in AML tumors. Hematopoietic stem cell (HSC)-like, granulocyte-macrophage progenitor (GMP)-like and monocyte-like AML cells are the main AML cellular hierarchies, which correlate with mutation, prognosis, and immunomodulation [41]. Another group used scRNA-seq data and ML-based approaches to define distinct transcriptomic states in Sézary syndrome as a type of cutaneous T-cell lymphomas. As a result, they characterized a gene signature that predicted the disease stage with high accuracy and found *FOXP3* (forkhead box protein P3) as the most important factor in the gene signature [28].

Cells from the same tumor may possess various mutations and exhibit distinct states. It is well known that copy number variations (CNVs) are a source of ITH [42]. However, the identification of CNVs from transcriptome data is difficult, especially for scRNA-seq data. Currently, several algorithms have been used to estimate CNVs from scRNA-seq data, such as inferCNV (a hidden Markov model-based approach) [43], HoneyBADGER (a hierarchical Bayesian model-based approach) [44] and CopyKAT (an integrative Bayesian segmentation approach) [45]. In attempts to define inter- and intratumor heterogeneity in patients with advanced non-small cell lung cancer (NSCLC), the inferCNV method was performed. The results showed that lung squamous carcinoma (LUSC) has significantly higher inter- and intratumor heterogeneity than LUAD [46]. Apart from lung cancer, by comparing primary tumors from different patients with epithelial OV through scRNA-seq, cellular heterogeneity was comprehensively characterized [47]. Distinct CNV trends in the same chromosomal region were observed in different patients, whereas they were approximately consistent in the same patient, demonstrating both intertumoral heterogeneity and inter-lesional consistency. Lineage plasticity, enabling the switch of cells from one phenotypic state to another, has been proposed as a source of ITH in cancer [48]. In primary NSCLC, both CNV and mitochondrial mutation-based lineage tracking analyses based on scRNA-seq data clearly showed that mixed-lineage and single-lineage tumor cells in the same patient originated from common tumor ancestors rather than from different origins [49]. Moreover, patients harboring high mixed-lineage features of different cancer subtypes had shorter survival. In addition, a mixed-lineage tumor cell-related gene, *AKR1B1* (Aldo-keto reductase family 1 member B), was speculated to be one of the master regulators of lineage plasticity and could serve as a candidate therapeutic target for NSCLC patients showing mixed-lineage features.

To clarify the formation mechanism of ITH, the tumor evolutionary dynamics of ITH were analyzed in a mouse colorectal cancer model by combining scRNA-seq with single-cell exome sequencing. The study revealed that new subpopulations emerge in part due to adaptation to changes in the microenvironment [50]. Moreover, Ma et al. developed a consensus clustering algorithm for scRNA-seq analysis to determine tumor cell states in liver tumors and found three major branches. Through analyzing conserved genes of different branches, *SPP1* (secreted phosphoprotein 1) was identified as the top conserved gene. Its encoded protein osteopontin, as a candidate regulator, drives ITH with the purpose of adapting the microenvironment for survival [51].

Cancer progression involves the acquisition of stem cell-like features. M. Malta et al. utilized a one-class logistic regression to extract transcriptomic feature sets from pluripotent stem cells and established stemness indices mRNAsi, which could be used to indicate molecular and clinical features in TCGA tumors. Moreover, mRNAsi is higher in tumor metastases and can reveal ITH at a single-cell resolution [52]. Another group used mRNAsi to divide glioblastoma (GBM) patients into two subtypes that had distinct overall survival and immunotherapy efficacy, suggesting its potential clinical application value [53].

### 3.2. Tumor Microenvironment

The tumor microenvironment (TME) is a complex and heterogeneous milieu, containing diverse cell populations. As an important parameter, the TME impacts the antitumor response and the efficacy of immunotherapy [54]. Thus, a deeper understanding of the TME is necessary to identify immune modifiers of cancer progression and develop immunotherapeutic strategies. However, the TME information is masked by bulk RNA-seq in tumor tissues. Thanks to computational algorithms, they offer solutions to infer cell populations from bulk tumor transcriptomic profiles. These algorithms fall into two main categories: one is a gene signature-based approach (e.g., ESTIMATE, which is based on the ssGSEA algorithm [55]), and the other is deconvolution-based approaches (e.g., CIBERSORT, which is based on linear support vector regression [56]). Recently, DL methods were utilized for cell deconvolution, showing great performances in inferring cellular composition of the tumor microenvironment compared to conventional ML-based approaches. For instance, Menden et al. developed a deep neural network (DNN) based framework, namely Scaden [57]. It outperforms existing deconvolution-based approaches in robustness and precision. We expect that DL-based methods will be a mainstay for cell deconvolution in the future.

Increasing evidence indicates that immune cell infiltration affects the prognosis of multiple cancers. Thus, many novel immune-related prognostic markers based on bulk RNA-seq data were separately established in various types of cancer by using ML algorithms such as LASSO [58], the Cox regression model [59], SVM [60], linear SVM [61], and principal component analysis (PCA) [62]. However, single type cohort training has the shortcomings of a small sample size and ITH of patients. To overcome these limitations, Thedinga et al. applied the extreme gradient boosting (XGBoost) tree to bulk transcriptome profiles from 25 kinds of cancer types to predict patient survival and utilized a network propagation algorithm based on a random walk with restart method to predict the TME status of patients [63].

The applications of scRNA-seq in dissecting the TME have provided new insights into the immune mechanism of response and resistance to immunotherapy. ICIs significantly improved overall survival and showed a favorable safety profile compared with chemotherapy in esophageal cancer (EC) patients [64]. Chen et al. identified a list of tumor-specific genes and four malignant signatures from scRNA-seq, which are potential novel markers for EC patients. Specifically, lymphocyte activation gene-3 (LAG3) may be a potential target for immunotherapy [65]. Furthermore, by combining the Assay for Transposase-Accessible Chromatin using sequencing (ATAC-seq) data with RNA-seq data of EC patients, Li et al. uncovered the potential transcriptional regulation characterization and constructed a LASSO-based prognosis-related subtype classifier, which is associated with TME heterogeneity and immunotherapy responses [66].

The efficacy of immunotherapy is correlated with the contents and types of T cells. Thus, T cell-related features are predictive of immunotherapy response. Guo et al. [67] performed scRNA-seq for approximately ten thousand T cells from 14 patients without treatment to observe heterogeneity within various types of T cells. In addition, their study found that *IL1R2* (interleukin 1 receptor type 2), an activated tumor regulatory T-cell-related gene, was associated with poor prognosis, providing a novel biomarker for NSCLC patient stratification. Mei et al. [68] carried out scRNA-seq to reveal distinct immune cell compositions in patients with CRC and identified several clusters of T cells that enable patients to improve the response rate to ICIs, such as *CXCL13*^+^ and *XCL1/2*^+^ T cells. Recently, an integrative analysis of scRNA-seq and the T-cell repertoire in patients with different types of cancer found clonotypic expansion of effector-like T cells both in tumor and normal adjacent tissue, and showed that patients with a gene signature of clonotypic expansion of effector-like T cells respond better to ICI therapy [69]. In addition, Caushi et al. revealed that CD8^+^ T cells specific for mutation-associated neoantigens had a higher checkpoint score and exhaustion score in patients without a major pathologic response, which contributed to ICI resistance [70].

Apart from T cells whose contents and types have effects on ICI therapy, other cell populations that correlate with the response to ICI have been determined recently [71]. Increasing evidence also indicates that cancer-associated fibroblasts (CAFs) have an influence on the TME and immunotherapy sensitivity. Lu et al. constructed a CAF score model by using RF and LASSO algorithms, which can be used to accurately predict infiltrating immune cell abundance and response to immunotherapy [72]. Moreover, they found that FGFR4 (fibroblast growth factor receptor), as a CAF-related molecule, may play an oncogenic role in pancreatic cancer. Yang et al. constructed an ML predictive model based on ligand-receptor gene pairs between macrophages and tumor cells. It was associated with the prognosis of LUAD patients [73].

It is critical for understanding cancer to determine cell states and multicellular communities. Previous studies have uncovered many phenotypic classes in human tumors, such as T-cell infiltration. However, these classifications oversimplify the TME information. Thus, researchers developed EcoTyper, an ML framework, for the large-scale identification of cell states and communities from bulk tissue samples [15]. Specifically, by applying EcoTyper, they found many novel cell states that were not previously identified by scRNA-seq. Moreover, the majority of cell states are significantly related to prognosis. Interestingly, AEBP1^+^ foamy macrophages were associated with a shorter survival time, suggesting that they could be an immunotherapeutic target in cancer. In addition, EcoTyper can reconstruct multicellular ecosystems and identify cohesive cellular communities. They also applied it to diffuse large B cell lymphoma (DLBCL) tumors [74].

### 3.3. Immunotherapy Toxicity

Despite great clinical benefits from cancer immunotherapy, an increasing number of patients with cancer develop unwanted side effects, including irAEs [8]. However, the immunological mechanisms responsible for irAEs remain poorly understood. To elucidate the pathogenesis of irAEs, scRNA-seq has been frequently utilized to identify the changes in immune cell compositions and effector programs underlying irAEs. In particular, single-cell analysis found that CXCR3^+^CD8^+^ effector memory T (T_EM_) cells were involved in both response and irAEs in anti-PD-1-treated patients with hepatocellular carcinoma (HCC). Further analysis identified distinct TNF interactions between CXCR3^+^CD8^+^ T_EM_ cells and myeloid cell populations specific to response and irAEs. Distinct TNF pathways could be harnessed to uncouple the response from irAEs in patients with HCC who received ICI therapy. Intriguingly, selective TNFR2 inhibition can improve the efficacy of ICIs without exacerbating irAEs, highlighting its potential as a promising combination strategy to uncouple antitumor efficacy and immunotherapy toxicity [75]. MicroRNA-146a (miR-146a) has been proposed as a molecular target for preventing ICI-mediated irAEs, which participates in regulating autoimmune dysregulation caused by ICIs [76]. The scRNA-seq data showed that mice lacking miR-146a exhibited increased T cell activation and neutrophil numbers, indicating that it is a potential molecular target. A spectacular accumulation of cytotoxic and proliferative CD8^+^ T cells was observed in ICI-induced colitis, with no significant depletion of regulatory T cells (Tregs) [77]. Further analysis revealed that the mTOR pathway was activated in infiltrated CD8^+^ T cells in colitic lesions, and mTOR blockade can inhibit T-cell infiltration and synergistically suppress tumor growth with an anti-PD-1 antibody [78]. Furthermore, researchers found that napsin A, as a self-antigen, elicited strong CD8^+^ T cell responses, which may promote irAEs [79].

Similar changes in T-cell subclusters were observed in other irAEs induced by different ICIs, such as myocarditis, hepatitis, and arthritis. An expansion of CD45RA^+^ effector memory CD8^+^ T cells was found in myocarditis patients exposed to ICI agents [80]. Moreover, a study showed that chemokine and metabolic pathways were altered by anti-PD-1 therapy, which may contribute to cardiac damage [81]. Additionally, PDK4 (pyruvate dehydrogenase kinase 4) was upregulated in the hearts of animals treated with a PD-1 antagonist, suggesting that it could serve as an early marker to identify cardiac irAEs. ICI-mediated hepatitis is related to an activated immune signature in all subtypes of T cells and T helper 1 polarization [82]. Specifically, the depletion of CD8^+^ T cells eliminated ICI-mediated hepatitis, highlighting the pivotal role of CD8^+^ T cells in ICI-induced liver injury. Furthermore, transcriptomic analysis revealed the existence of close interactions between monocyte-derived macrophages and T cells, indicating that monocyte-derived macrophages participate in the development of ICI-induced hepatitis. Arthritis, as the most common form of rheumatic irAEs, greatly impairs quality of life and even leads to permanent discontinuation of ICI therapy. To decipher the mechanisms of arthritis induced by ICIs, 5′ end scRNA-seq was performed to depict the states of immune cells in peripheral blood and synovial fluid samples from patients with arthritis-irAE [83]. The results revealed that the Th1-CD8^+^ T axis has important implications for arthritis-irAE pathogenesis. Myeloid cell populations can secrete chemokines to recruit effector CD8^+^ T cells into joints, indicating their critical roles in the pathogenesis of ICI-mediated arthritis. Notably, Tregs were enriched in the synovial fluid of patients with arthritis-irAE, possessing heightened suppressor functions. In fact, another study suggested that Treg function might be negatively correlated with the severity of irAEs, indicating heterogeneous functions of Tregs in irAEs [84]. Tregs from melanoma patients with irAEs also presented intense transcriptomic reprogramming, which is consistent with the Treg dysfunction observed in patients with autoimmune diseases [85]. The biological role of Tregs in arthritis-irAE needs further exploration.

In addition to T cells, other cell types have also been demonstrated to contribute to the development of irAEs. The early changes in B cells following ICI treatment were associated with both the timing and frequency of irAEs, suggesting that targeting B cells may alleviate toxicities in these patients [86]. Another team revealed that liver-resident Kupffer cells drove neutrophil-mediated liver toxicity in the setting of CD40 agonist immunotherapy. Kupffer cells respond to lymphocyte-derived IFN-γ, subsequently producing IL-12 and ultimately prompting a neutrophil response [87].

### 3.4. Drug Resistance and New Target Discovery

AI-based transcriptomic analysis could also hint at the importance of some novel targets in immunotherapy response (Table 1). Bareche et al. established a gene expression signature (PredictIO), showing its superior predictive value over other biomarkers [88]. Furthermore, they combined TIDE, which integrated the expression signatures of T-cell dysfunction and exclusion to model tumor immune evasion [89], with PredictIO to identify potential targets to overcome ICI resistance. As a result, F2RL1 (protease-activated receptor-2 like trypsin receptor 1) and RBFOX2 (RNA binding fox-1 homolog 2), with high T-cell dysfunction scores, were regarded as novel biomarkers and potential targets. Another case is that researchers confirmed the significance of SAA2 (serum amyloid A2) and CFB (complement factor B) in prognosis and therapeutic response by using ML algorithms, suggesting that these two proteins play important roles in tumor metastases and can be predictive biomarkers [90].

Currently, numerous prediction models have been constructed using ML to predict ICI response [92,94,95]. Numerous studies have concentrated on correlative observations between immune-related gene expression and immunotherapy responses in various cancers, while very little is known about its role in antitumor immunity, which is important for target identification. ScRNA-seq technology could accelerate new target discovery by deepening the understanding of the underlying immune response mechanism and identifying new targets involved in tumor initiation, progression, metastasis, and immune escape. For example, Somani et al. found that interleukin-1 receptor associated kinase-4 (IRAK4)-mediated NF-κB activity positively correlated with T cell exhaustion in pancreatic ductal adenocarcinoma (PDAC). The loss or inhibition of IRAK4 abrogated checkpoint molecule expression and elevated the infiltration abundance and activity of infiltrative CD4^+^ and CD8^+^ T cells. The results from scRNA-seq analysis further revealed that IRAK4 inhibition induced myeloid and fibroblast reprogramming toward acute inflammatory responses, resulting in a T cell supportive TME [96]. Furthermore, IRAK4 inhibition synergized with ICI treatment to reprogram the TME and improve the outcome achieved in a mouse model, suggesting a translatable therapeutic strategy to overcome immunotherapy resistance. In another case, Li et al. [97] found that NKG7 (natural killer cell granule protein 7) expression was specifically expressed by CD8^+^ T cells and natural killer (NK) cells based on scRNA-seq datasets and observed that the efficacy of ICIs was strikingly reduced in *NKG7*-deficient mice.

To explore the transcriptomic effects of anti-PD-L1 and anti-TGF-β therapy on tumor and TME compartments, scRNA-seq was used to profile the transcriptional changes induced in a mouse model after PD-L1/TGF-β blockade. The results showed that C-C motif chemokine ligand 5 (CCL5) may be a potential target for enhancing immune cell infiltration [98]. ScRNA-seq analysis revealed substantial heterogeneity within early-stage LUAD harboring EGFR mutations and identified a crucial modulator, E74-like ETS transcription factor 3 (ELF3), in modulating the interactions among tumor cells and TME components, suggesting its potential as a therapeutic target in LUAD [99]. To explore the mechanisms of myeloid-targeted immunotherapy, Zhang et al. performed scRNA-seq analyses on immune and stromal populations from CRC patients and murine tumor models, identified distinct myeloid populations that convey differential sensitivity to CSF1R blockade, and defined active immune responses in dendritic cells and T cells treated with a CD40 agonist antibody [100]. In addition, Gao et al. found that CRC patients with the microsatellite stability phenotype exhibited more macrophage infiltration and protein tyrosine phosphatase nonreceptor type 11 (SHP2) phosphorylation than patients with the microsatellite instability-high phenotype, implicating SHP2 as a potential target for immunotherapy [101].

ITH confers drug resistance to immunotherapy, resulting in diverse responses to therapy. Using scRNA-seq, researchers found that the CD44^+^ cancer stem cell population may induce drug resistance in patients with HCC [102]. Li et al. identified a unique CD8^+^ T-cell subset with high levels of oxidative phosphorylation in melanoma patients by scRNA-seq analysis, which correlated with ICI resistance [103]. Another specific cell subpopulation with immunotherapy persistence, namely immunotherapy persister cells (IPCs), has also been identified by scRNA-seq and was found to be dependent on baculoviral IAP repeat-containing protein 2 (Birc2) and Birc3 [104]. Interestingly, it has been reported that BIRC2 impairs anticancer immunity and immunotherapy efficacy [105]. Thus, the combination of ICIs and BIRC2 inhibitors provides a potential strategy to overcome immunotherapy resistance. Furthermore, Chen et al. proposed a deep transfer learning (DTL) framework, called scDEAL, to predict drug responses [16]. The DTL model integrated large-scale bulk and single-cell transcriptome data using a domain-adaptive neural network (DaNN) algorithm to predict cancer drug response at the single-cell level. It performed well on six drug-treated scRNA-seq datasets with validated drug-response labels. A similar model was established to infer the cancer drug response by the DNN algorithm, namely Precily [93]. These bioinformatics frameworks could be used to predict the immunotherapy response and resistance of individual cells.

## 4. Conclusions and Future Perspectives

Transcriptomic technologies are developing rapidly, and have greatly accelerated research on cancer immunotherapy. Recent advances in multi-omics technologies have expanded the application scope of transcriptome technologies. Multi-modal data provide comprehensive molecular profiles, enabling a more complete understanding of cancer biology. Furthermore, single-cell spatial transcriptomics can capture spatial distribution information and reveal intercellular communication acting in situ [106]. In addition, with the development of AI technology in biomedical research, integrating state-of-the-art computational methods into high-dimensional single-cell analysis (e.g., transcriptomics, proteomics, epigenomics) contributes to research on cancer immunotherapy [91]. With the advent of the era of precision medicine, AI-assisted transcriptome technologies will be continuously developed and more frequently used in immunotherapy research and will provide valuable information for the development of new strategies to improve the efficacy of cancer immunotherapy.

## Figures and Tables

**Table 1:** Application of AI approaches in transcriptomic analysis.

Input Data	AI Model	Tumor	Function	Application	Ref.
Bulk RNA-seq data	ML: Innovative one-class logistic regression	Pan-cancer	Indicating prognosis, molecular subtypes, and immunotherapy efficacy	ITH	[52]
ScRNA-seq data	ML: RF	Acute myeloid leukemia	Distinguishing cell types in the acute myeloid leukemia ecosystem	ITH	[40]
ScRNA-seq data	ML: Consensus clustering	Hepatocellular carcinoma and Intrahepatic cholangiocarcinoma	Determining tumor cell states	ITH	[51]
Spatial RNA-seq data and images	DL: CNN	Breast cancer	Linking gene expression with visual features in cell morphology	ITH	[91]
Bulk RNA-seq and ICB response data	ML: NMF	Syngeneic cancer cell lines and mouse model across 13 cancer types	Reliably stratifying patients regarding ICB response	ITH	[34]
Bulk RNA-seq, scRNA-seq and drug response data	ML: RF	Ovarian cancer	Guiding identification of safe and effective combinatorial treatment regimens	Drug resistance	[92]
Bulk RNA-seq and mutations data	ML: SVM	12 cancer types in TCGA	Discriminating tumors with heterogeneous cell populations	ITH	[33]
ScRNA-seq data	DL: Denoising autoencoder	Malignant melanoma	Addressing a substantial amount of noise	ITH	[23]
ScRNA-seq data	ML: k-TSP	Various types of cancer	Classifying a single cell type and minimizing the batch effects	ITH	[26]
Bulk RNA-seq data	DL: ELM	Gastric cancer, ovarian cancer, and so on	Identifying cancer molecular subtypes that are much more clinically relevant	ITH	[24]
Bulk RNA-seq and scRNA-seq data	DL: DNN	550 cancer cell lines	Inferring treatment response in cancers	Drug resistance	[93]
ScRNA-seq data	DL: GAN, deep autoencoders	Colorectal cancer	Identifying the batch-specific cell types	ITH, TME	[27]
Bulk RNA-seq data	ML: NMF	Ovarian cancer	Supporting the classification of new model systems of ovarian cancer subtypes	ITH	[35]
Bulk RNA-seq and scRNA-seq data	DL: DaNN	Various types of cancer	Predicting drug responses in scRNA-seq	ITH, drug resistance	[16]
Bulk RNA-seq, scRNA-seq and spatial RNA-seq data	ML: EcoTyper	16 types of carcinoma	Providing a framework for large-scale profiling of cellular ecosystems in any tissue	TME, ITH	[15]
Bulk RNA-seq data	ML: SVM	33 tumor types from TCGA	Confirming the classification of inflammasome	TME	[60]
Bulk RNA-seq data	ML: linear SVM	Gastrointestinal cancer	Predicting immunotherapy response in patients	TME	[61]
Bulk RNA-seq data	ML: XGBoost, RWR	25 tumor types from TCGA	Predicting the TME and immune states of patients	TME	[63]
Bulk RNA-seq and scRNA-seq data	ML: XGBoost	Lung adenocarcinoma	Predicting the prognosis of ligand–receptor gene pairs	TME	[73]
Bulk RNA-seq data	ML: RF, LASSO	Pancreatic cancer	Predicting the abundance of immune cell infiltration and the response of immunotherapy	TME	[72]
Bulk RNA-seq and scRNA-seq data	ML: PCA, RF	12 tumor types, melanoma	Predicting response to ICIs	Drug resistance	[88,94]
Bulk RNA-seq data	ML: LASSO	Urothelial carcinoma	Estimating ICI response and prognosis of patients	Drug resistance	[95]
Bulk RNA-seq data	ML: RSF, PCA	Renal cell carcinoma	Predicting tumor progression and relapse	Target discovery	[90]

Abbreviations: ITH, intratumor heterogeneity; TME, the tumor microenvironment; ScRNA-seq, single-cell RNA sequencing; RF, random forest; CNN, convolutional neural network; NMF, nonnegative matrix factorization; SVM, support vector machines; k-TSP, k-top scoring pairs; ELM, extreme learning machines; DNN, deep neural network; GAN, generative adversarial network; DaNN, domain-adversarial training of neural networks; XGBoost, the extreme gradient boosting; RWR, random walk with restart; LASSO, least absolute shrinkage and selection operator; RSF, random survival forest; PCA, principal component analysis.

## Data Availability

Data sharing not applicable.
